# COMPARING OLDER ADULTS’ EXPOSURE TO AND SHARING OF HEALTH-RELATED MESSAGES ON FACEBOOK BY CHRONIC CONDITION STATUS

**DOI:** 10.1093/geroni/igac059.2464

**Published:** 2022-12-20

**Authors:** Matthew Schroeder, Carrie Miller, Sherry Pagoto

**Affiliations:** Regenstrief Institute, Inc., Indianapolis, Indiana, United States; VCU Fuemmeler Lab, Richmond, Virginia, United States; University of Connecticut, Storrs, Connecticut, United States

## Abstract

We compared older adult Facebook users with and without a chronic health condition on their frequency of exposure to and posting health-related messages. Demographics, social media use, and chronic condition status were collected via survey. Regular Facebook users aged 50+ years were recruited via Qualtrics. Participants reported if they had seen, posted, or shared: health-related information; about others’/their own health behaviors (e.g., exercise); and about others’/their own chronic condition. Responses were dichotomized as “Rarely” or “At least once a month”. Six logistic regression models, controlling for demographics and Facebook login frequency, assessed whether viewing and/or posting health-related messages differed by chronic condition status. Respondents (N=697; 77.9% female; 87.9% non-Hispanic White) were on average 61.2 years old (SD=7.9). One-half reported a chronic condition (n=351; 50.4%). In adjusted models, those with a chronic condition had a higher likelihood of seeing posts from others with health information (OR=1.37; 95% CI: 1.01, 1.86) and about others’ health conditions (OR=1.64; 95% CI: 1.20, 2.23) ≥ monthly (vs no chronic conditions). Similarly, those with a chronic condition had a higher likelihood of posting or sharing health information (OR=1.52; 95% CI: 1.03, 2.24) and about their chronic condition (OR=1.93; 95% CI: 1.16, 3.21) ≥ monthly. People with and without chronic conditions did not differ in how often they saw or posted about health behaviors. Older adults with chronic conditions were more likely than those without chronic conditions to regularly see and share health information on Facebook. The content and accuracy of this health information should be explored.

